# Supplementary material for: Unglycosylated Soluble SARS-CoV-2 Receptor Binding Domain (RBD) Produced in E. coli Combined with the Army Liposomal Formulation Containing QS21 (ALFQ) Elicits Neutralizing Antibodies against Mismatched Variants
Source: Vaccines (Basel). 2022 Dec 25;11(1):42. doi: 10.3390/vaccines11010042 (PMC9864931; doi:10.3390/vaccines11010042)
Supplement: Supplementary file 1 [file vaccines-11-00042-s001.zip › vaccines-2080742-supplementary.pdf]

Supplementary Figure S1

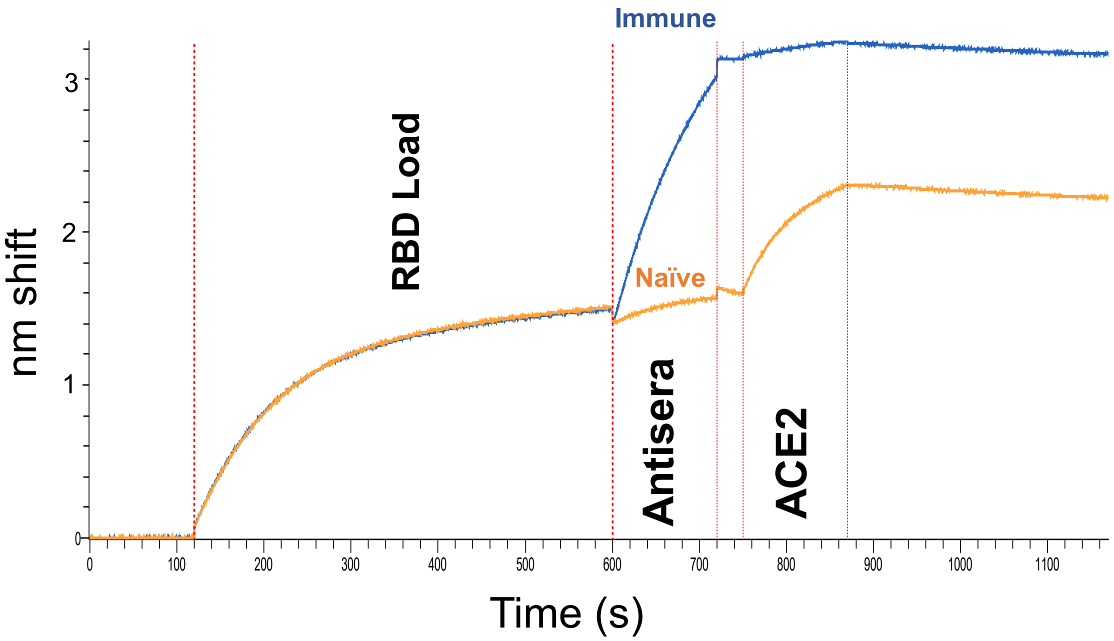

Supplementary Figure S2

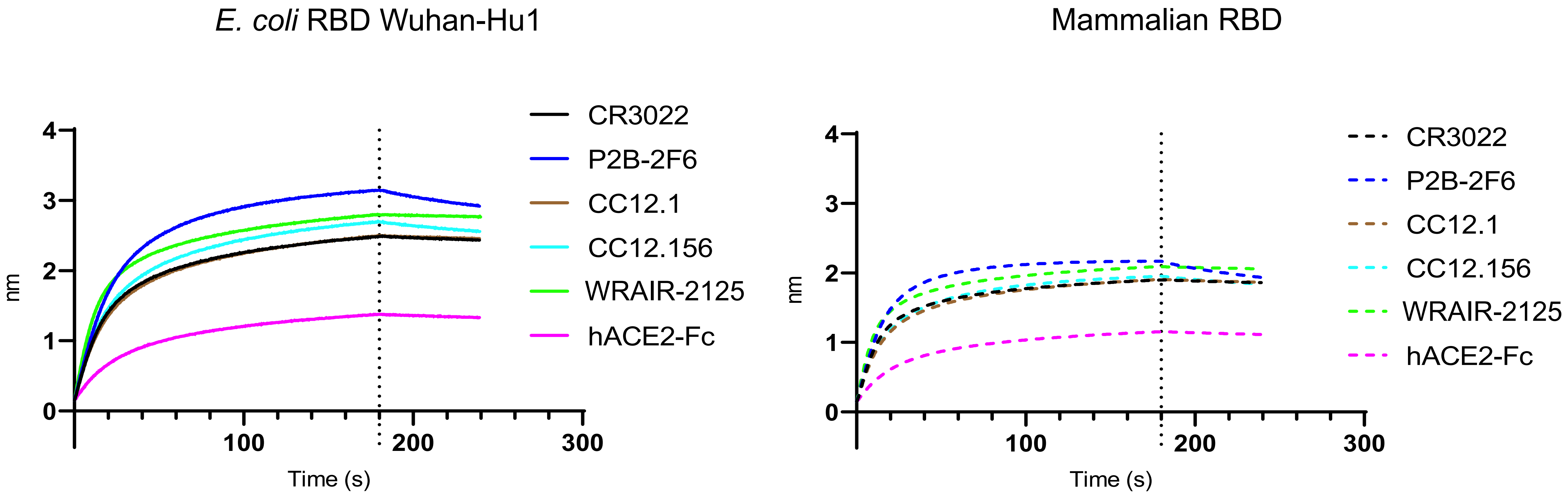

Supplementary Figure S3

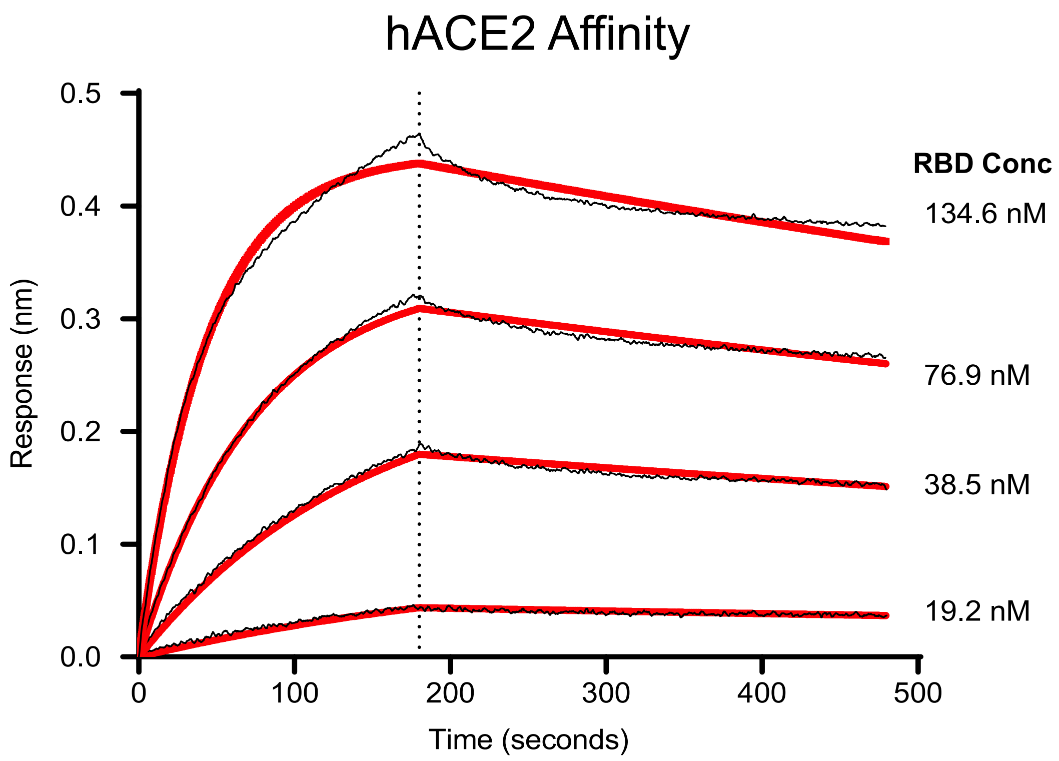

K_D (nM)	K_D error	k_{on} (1/M * s)	k_{on} error	k_{dis} (1/s)
3.50	4.15E-11	1.65E+05	8.71E+02	5.77E-04

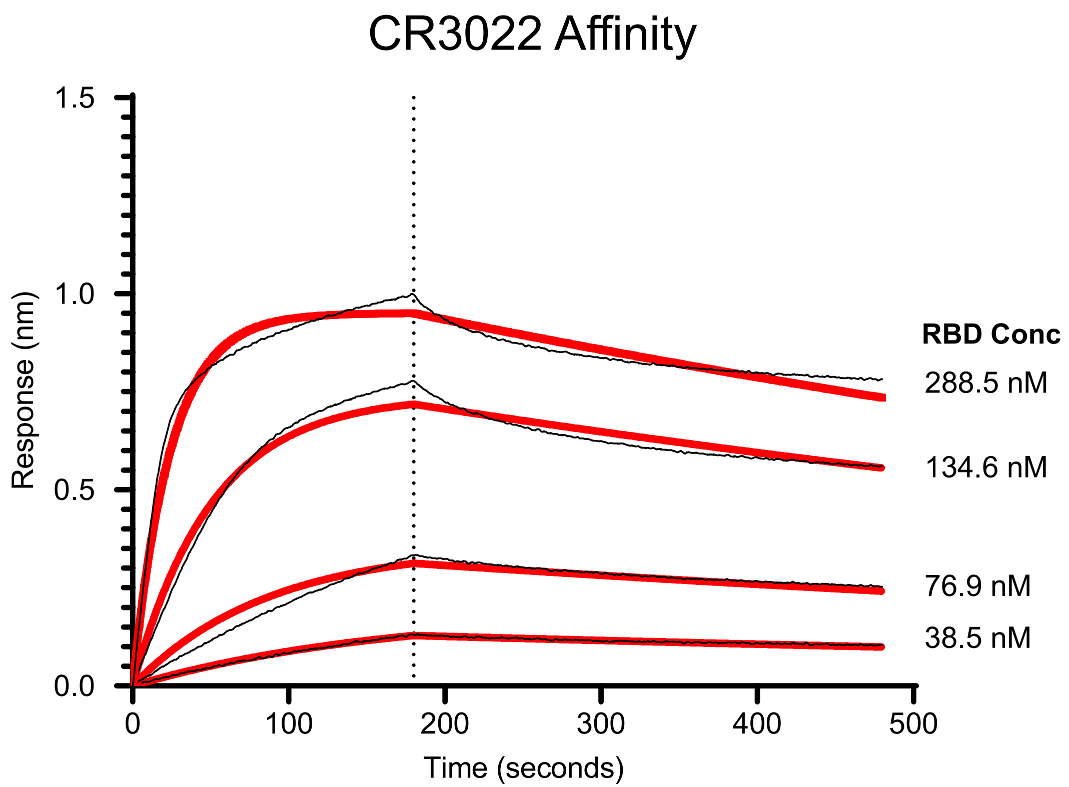

K_D (nM)	K_D error	k_{on} (1/M * s)	k_{on} error	k_{dis} (1/s)
6.13	8.04E-11	1.40E+05	1.06E+03	8.56E-04
